# Effects of Betahistine on Vestibulo-Ocular Reflex in Normal Healthy Adults: A Randomized Double-Blind, Placebo-Controlled Trial

**DOI:** 10.7759/cureus.26452

**Published:** 2022-06-30

**Authors:** Wan Ahmad Amiruddin Wan Hassan, Khairul Ridhwan Mohd Nasir Mohd Nasir, Saiful Adli Jamaluddin, Edre Mohammad Aidid, Yahia F Hussein Al-Hadeethi

**Affiliations:** 1 Department of Otorhinolaryngology-Head and Neck Surgery, Sultan Ahmad Shah Medical Centre @IIUM (International Islamic University Malaysia), Kuantan, MYS; 2 Department of Audiology and Speech-Language Pathology, Kulliyyah of Allied Health Sciences, International Islamic University Malaysia, Kuantan, MYS; 3 Department of Community Medicine, Kulliyyah of Medicine, International Islamic University Malaysia, Kuantan, MYS

**Keywords:** placebo, betahistine, video head impulse test, vestibulo-ocular reflex, vertigo

## Abstract

Introduction

Vertigo, or the perception of a spinning sensation, is a common symptom experienced by patients who are referred to Otorhinolaryngology clinics. Betahistine is a medication that has been widely used to treat vertigo and its accompanying symptoms. However, the effects of this medication on the vestibulo-ocular reflex (VOR) are still unknown. Initially, it was assumed that betahistine should be discontinued prior to any vestibular tests, particularly the video head impulse test (vHIT).

Method

Thirty young healthy adults were randomly divided into two equal groups for this randomized double-blind clinical study (betahistine 24 mg and placebo). Baseline pure-tone audiometry (PTA), tympanometry, and VOR measurements were taken, followed by experimental measurements at one hour, four hours, eight hours, and 24 hours after consumption. The video head impulse test (vHIT) was used to determine the VOR.

Result

Betahistine had no statistically significant effect on vestibulo-ocular reflex gain (F(4,140) = 0.601, p = 0.662). The gain variability across repetitive head impulses remained constant over time.

Conclusions

Betahistine has no effect on the vestibulo-ocular reflex. As a result, this medication can be taken prior to the vHIT procedure.

## Introduction

The American Association of Otolaryngology-Head and Neck Surgery Committee on Hearing and Equilibrium defines vertigo as “the sensation of motion when no motion is occurring relative to the earth's gravity, in contrast to motion intolerance, which is a feeling of dysequilibrium, spatial disorientation, or malaise during active or passive movement” [1]. A study in a specialized otology clinic in Malaysia in 2013 revealed that the prevalence of vestibular disorder is 32.2%, where the majority of patients were of Malay ethnicity [2]. According to Wong et al. (2022), the most common cause of vestibular disorder is benign paroxysmal positional vertigo (BPPV), which accounts for approximately 41%, followed by vestibular migraine (20.7%), Ménière's disease (5.8%), vestibular neuritis (3.3%), labyrinthitis (1.7%), and chronic vestibulopathy (0.8%) [3]. Antiemetics, histamine analogs, antihistamines, diuretics, steroids, antivirals, antimicrobials, calcium channel blockers, antidepressants, anticonvulsants, and aminopyridines are common drugs used to treat vertigo [4].

The most widely used antivertigo medication is betahistine [5]. It is the first-line treatment for vertigo, followed by cinnarizine and flunarizine [6]. According to Jeck-Thole and Wagner (2006), it has been used by approximately 130 million people since its inception [7]. Betahistine is commonly prescribed in hospitals to reduce the severity of vertigo/dizziness [4]. Betahistine is a D1 agonist and a D3 antagonist. This medication has been used to treat vertigo for many years. It has been shown to improve cochlear, vestibular, and cerebral blood flow [5,8,9]. Furthermore, by regulating capillary structures in the stria vascularis of the inner ear, betahistine can reduce pressure in the endolymphatic space and increase reabsorption of endolymphatic fluid [4,10].

According to Al-Tamimi et al. (2020), betahistine has also been used to reduce the severity and frequency of vertigo associated with Ménière's disease [11]. The recommended daily dose for adults is 24-48 mg in divided doses. Betahistine is metabolized in the liver to 2-pyridyl acetic acid (2-PAA) and excreted in the urine. Within 24 hours of ingestion, approximately 85%-91% will be detected in urine samples.

The stabilization of the eyes during head movement is referred to as the vestibulo-ocular reflex. According to history, Halmagyi and Curthoys pioneered the head impulse test in 1988 [12]. MacDougall et al. [13] developed a video method for testing the function of each of the six canals individually in 2009. The video head impulse test (vHIT) is a device that measures the gain of the angular vestibulo-ocular reflex (VOR) during head rotations based on the eye-to-head acceleration ratio [14,15]. This vHIT can detect covert and overt saccades, both of which are symptoms of vestibular hypofunction following head rotation. There are currently no published data on the effects of betahistine on vestibulo-ocular reflex gain in the Malaysian population. As a result, the purpose of this novel study was to assess the effects of betahistine on VOR in a normal healthy population using a commonly used daily dose of betahistine of 24 mg.

## Materials and methods

Study population

**Figure 1:**
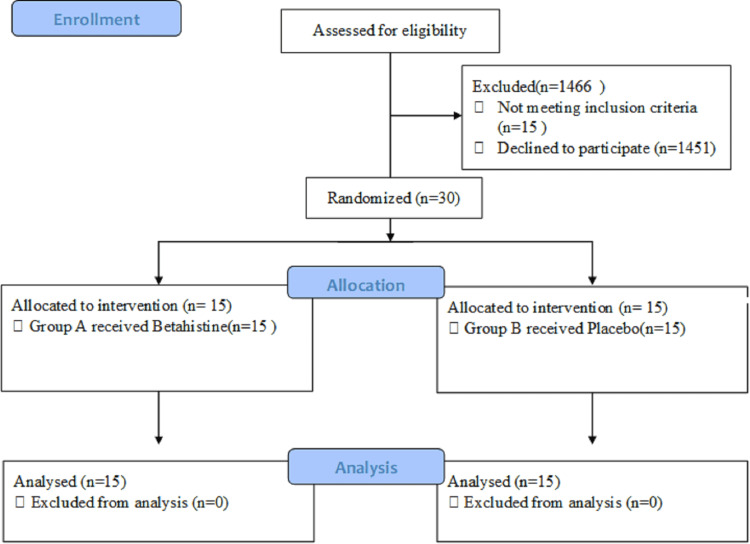
Study flowchart according to the Consolidated Standards of Reporting Trials (CONSORT)

This research was carried out in the balance room of the Sultan Ahmad Shah Medical Centre of International Islamic University Malaysia (IIUM)'s Department of Otorhinolaryngology-Head and Neck Surgery in Kuantan, Malaysia. The Sultan Ahmad Shah Medical Centre @IIUM had a total of 1,496 staff members working there. However, only 30 persons met the criteria to participate in the study, and they were randomly assigned to one of two groups. Group A received the betahistine, whereas group B received a placebo (Figure 1). This study included 30 adult healthy staff members, 13 males and 17 females ranging in age from 23 to 40 years old. All subjects had normal audiograms, tympanograms, and physical examinations, which included otorhinolaryngology exams.

Exclusion criteria for this study include (1) history of allergy to histamine analog group, primarily betahistine; (2) pregnant or lactating mother; (3) neck or spine problem; (4) psychiatric disorder or significant neurological diseases (e.g., depression, Parkinson's disease); (5) asthmatic; (6) renal or hepatic problem; (7) pheochromocytoma patient; and (8) peptic ulcer disease patient. This research was carried out in strict accordance with the principles of the Declaration of Helsinki. Each subject was given verbal and written information about the test procedure prior to testing. The risks and exclusion criteria are outlined, where signed consent is required. The project was approved by the International Islamic University Malaysia (IIUM) Research Ethics Committee (IREC), Kuantan, Pahang, Malaysia, which issued approval IREC-2021-137.

Methods

For the baseline, all subjects underwent a battery of tests, including otoscopy, pure-tone audiometry (PTA), tympanometry, and horizontal vHIT. Then, for each medication, the subjects were randomized using computer-generated randomization to generate blocks of patient numbers. When a subject was assigned a code, he or she was given a sealed envelope containing that subject's experimental group, which can only be opened by an appointed study nurse. The type of medication given was unknown to both the investigator and the subject. The participant took the prescribed medication at a specific time. VOR was measured experimentally at one hour, four hours, eight hours, and 24 hours after consumption of medication. The interval testing is done to determine the pharmacokinetics of betahistine. Betahistine plasma concentrations peaked one hour after ingestion, with a terminal elimination half-life between three and five hours. To avoid interexaminer bias, only one examiner performed the video head impulse test.

Otoscopy, Pure-Tone Audiometry, and Tympanometry

The Heine™ otoscope (HEINE Optotechnik GmbH & Co. KG, Gilching, Germany) was used for outer ear inspections to detect any abnormalities in the ear canal and outer ear. To measure and assess middle ear function, a baseline Grason-Stadler Tympstar™ Middle Ear Analyzer (Grason-Stadler Ltd., Maine, USA) was used (tympanometry test). This test can detect any middle ear problems based on the middle ear compliance measured. GSI Audiostar (Grason-Stadler Ltd., Maine, USA) and Interacoustics AC40 (Interacoustics A/S., Middelfart, Denmark) with supra-aural headphone were used to obtain their hearing thresholds for pure-tone audiometry (PTA). At Sultan Ahmad Shah Medical Centre @IIUM, pure-tone audiometry was performed in a soundproof room at 125, 250, 500, 1,000, 2,000, 4,000, and 8,000 Hz.

Video Head Impulse Test

The video head impulse test (vHIT) was performed using the EyeSeeCam™ vHIT system version 1.2 (Interacoustics A/S., Middelfart, Denmark) by using a lightweight goggle with an infrared camera to measure pupil movements and head movements. The subject was instructed to stand 1 m in front of a wall with a fixed target point at eye level. To reduce slippage, video-oculography (VOG) goggles were tightly fitted to the subject's head. The camera recorded the subject's eye movement while it was fixated on the target point. The researcher stood behind the subject and clasped three fingers beneath the patient's mandible and a thumb and forefinger above the jaw line. The subject's head was passively and randomly rotated to the right and left in an abrupt, concise, and unpredictable manner with a low amplitude (approximately 5°-15°) and a high peak velocity (150°/s-250°/s). Finally, the VOR gain for the lateral semicircular canal was calculated as a ratio of eye velocity to head velocity.

Statistical Analysis

In this study, the IBM Statistical Package for the Social Sciences (SPSS) version 26 (IBM Corps, New York, USA) was used for statistical analysis. Means and standard deviations for the time taken to consume betahistine or placebo, as well as the vHIT gain, for both the right and left vestibular systems were calculated and analyzed using the repeated measures ANOVA (RM-ANOVA) test. A p-value of less than 0.05 was regarded as significant.

## Results

**Figure 2:**
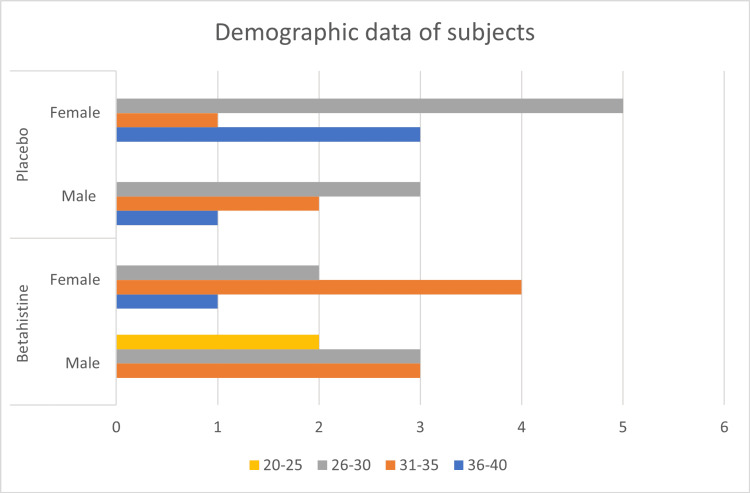
Distribution of subjects involved in the study

Thirty people (13 men and 17 women) between the ages of 23 and 40 years participated in the study. The average age was 30 years, with a standard deviation of four years. They were then divided into two equal groups (group A and group B). Group A consisted of 15 subjects (eight males and seven females) for betahistine, and group B consisted of 15 subjects for placebo (six males and nine females) (Figure 2).

**Figure 3:**
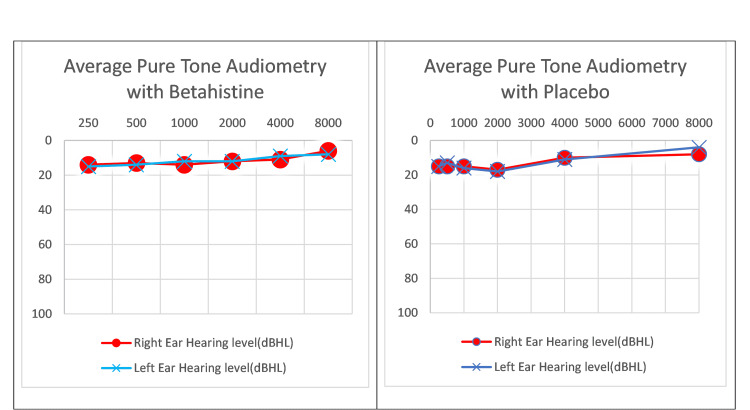
Average pure-tone audiometry of betahistine and placebo

Pure-tone audiometry (PTA) and tympanometry tests were performed on all subjects. The PTA test was passed by all subjects (Figure 3). However, for the tympanometry test, two subjects in the betahistine group had type Ad (suggesting highly compliant middle ear system) while three in the placebo group had type As (suggesting reduced middle ear compliance) and one had type Ad (suggesting highly compliant middle ear system).

**Figure 4:**
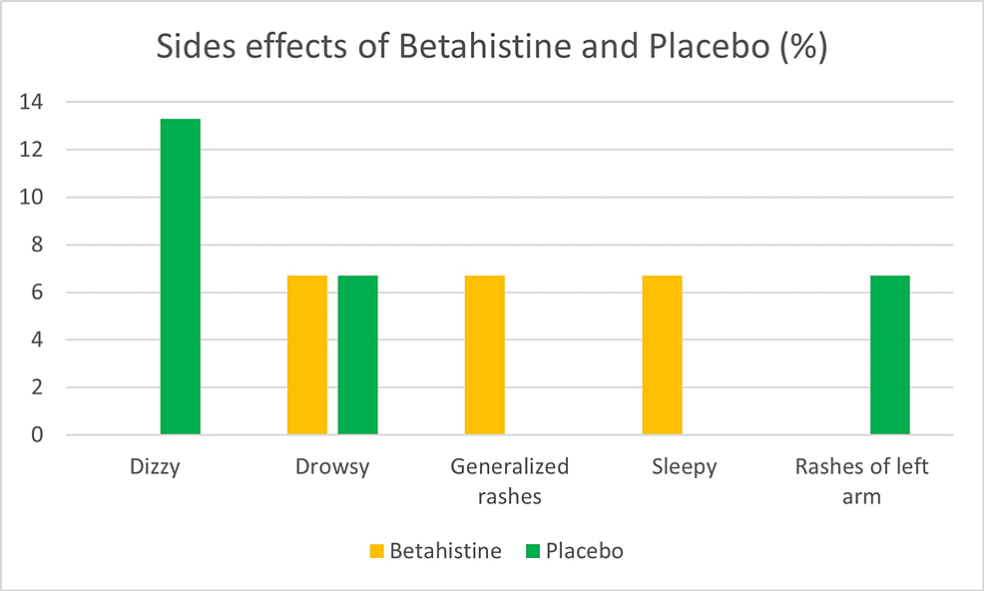
Side effects of betahistine and placebo

Drowsiness, generalized rashes, and sleepiness were the most common side effects in betahistine groups, accounting for approximately 6.7% of the total. In the placebo groups, the majority of subjects (13.3%) reported dizziness, followed by drowsiness and left arm rashes (6.7%) (Figure 4).

The mean VOR gain at baseline for right lateral semicircular canal (SCC) vHIT with betahistine ingestion was 0.95, one hour was 1.02, four hours was 1.03, eight hours was 1.03, and 24 hours was 1.02. The highest VOR gain occurred at four hours, and the lowest VOR gain occurred at 24 hours. The mean VOR gain at baseline for left lateral SCC vHIT with betahistine ingestion was 0.99, one hour was 1.08, four hours was 1.08, eight hours was 1.10, and 24 hours was 1.07. The highest VOR gain occurred at eight hours, and the lowest VOR gain occurred at 24 hours. 

**Figure 5:**
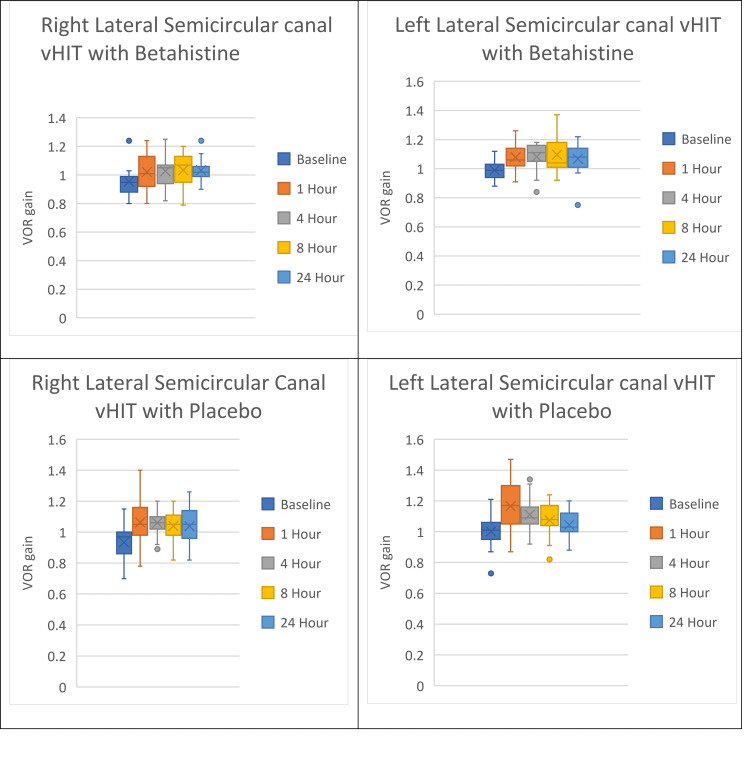
Effects of betahistine and placebo on lateral semicircular canal vHIT vHIT: video head impulse test, VOR: vestibulo-ocular reflex.

The mean VOR gain at baseline for right lateral SCC vHIT with placebo ingestion was 0.93, one hour was 1.06, four hours was 1.06, eight hours was 1.04, and 24 hours was 1.04. The highest VOR gain occurred at one hour, and the lowest VOR gain occurred at the 24-hour baseline. The mean VOR gain at baseline for left lateral SCC vHIT with placebo ingestion was 1.00, one hour was 1.17, four hours was 1.11, eight hours was 1.07, and 24 hours was 1.04. The highest VOR gain occurred at one hour, and the lowest VOR gain occurred at the 24-hour baseline (Figure 5). The repeated measures ANOVA test was used to analyze the authors’ study. Betahistine had no statistically significant effect on vestibulo-ocular reflex gain (F(4,140) = 0.601, p = 0.662).

## Discussion

Before beginning the battery of tests, patients were recommended to stop taking betahistine, as well as any other medications or treatments for vertigo. The purpose of this research is to investigate the influence that betahistine has on the amount of gain in the vestibulo-ocular reflex (VOR). The VOR increase over time was unaffected by the administration of a single dose of 24 mg.

In this work, the comparison was made between the right and left VOR gain that occurred after the use of betahistine. Betahistine consumption resulted in an increase in VOR gain with time in the right lateral semicircular canal (LSCC) vHIT at one and four hours, but this gain was reduced at eight hours and 24 hours. Left LSCC vHIT after the consumption of betahistine, on the other hand, displays a rise in VOR gain at one and eight hours, but a decrease at four and 24 hours. At one, four, and eight hours, betahistine induced a greater overall VOR gain; however, at 24 hours, this effect was attenuated. This research is different from that conducted by Kingma and colleagues in 1997 [16]. According to their study on the dose-dependent effects of betahistine on VOR, the greatest decrease in gain occurred approximately four hours after the administration of the 16 mg dose during the torsion swing experiment and the 32 mg dose during the head-shaking experiment. They carried out three tests, which included a low-frequency rotation in the dark, the visuo-vestibular interaction test (VVIT), and a high-frequency passive head rotation. The first two examinations consisted of rotating the patient while they kept their eyes open in the darkness. Patients participated in the high-frequency passive head rotation while wearing a helmet equipped with a torque motor that oscillates the head. The procedure was carried out in the light. They concluded that there were no impacts of VOR with betahistine during low-frequency torsion swing stimuli in the dark. However, they found a considerable drop of VOR gain up to 50% at four and six hours after drug intake, with the highest dose at 32 mg. Their weakness was that they relied on head thrust with a low frequency, whereas our study relied on head thrust with a fast speed. It is possible that the difference in the findings of this work is attributable to the fact that this work only employed a single dose of betahistine, which was 24 mg. In addition, rather than patients who might have a unique response to betahistine, this study opted to select healthy volunteers for participation.

The utilization of cutting-edge vHIT technology in conjunction with high-speed testing was beneficial in this study. The vHIT is able to evaluate the function of the VOR on high-frequency head thrust. Traditional vestibular tests, such as the caloric test and the rotatory chair, may be rendered obsolete by vHIT, as suggested by Morrison et al. (2022) [17]. Aside from that, the purpose of using healthy people is to monitor the typical changes in a physiological state that are brought on by the administration of drugs.

Furthermore, the limitations of the trial in this research are recognized, which included a modest dose of betahistine (24 mg), a small sample size, and the use of healthy participants. This research is comparable to those of Patel et al. (2014) [18] and Gordon et al. (2003) [19] in terms of the overall outcome of the study, whereby no effect of medication was found on the vestibulo-ocular reflex. Patel et al. examined the impact of prochlorperazine on normal vestibular ocular and perceptual responses in a placebo-controlled, randomized, double-blind crossover research. In comparison to placebo, prochlorperazine had no effect on any measure of nystagmus or perceptual vestibular function [18].

On the other hand, Gordon et al. [19] evaluated VOR using the sinusoidal harmonic acceleration (SHA) test. They concluded that there were no significant differences between treatments (betahistine vs. placebo) for any of the tested frequencies and that a single dose of betahistine 48 mg had no effect on low-frequency VOR parameters or psychomotor performance while having a marginally significant effect on seasickness in rough seas. This is not the case in this investigation, as we employed vHIT to evaluate the VOR with a single 24 mg dose of betahistine. Betahistine was only found to have very mild adverse effects in clinical trials. Drowsiness, generalized rashes, and tiredness were some of the adverse reactions that occurred in three of the 15 test individuals, which is around 20%. This finding is in agreement with the findings of Strupp et al. (2008) [20], who discovered that betahistine is a safe medication with only mild adverse effects.

## Conclusions

For normal healthy individuals, the use of 24 mg of betahistine shows that the VOR gain in high-speed head thrust is unaffected by the drug's presence. As a result, VOR tests specifically high-frequency head thrusts can be conducted without disrupting the consumption of betahistine. Additional research is required to assess the effects of VOR in patients who were administered a high dose of betahistine (48 mg) inclusive of patients with dizziness as subjects as opposed to only healthy individuals. Additionally, a longitudinal study on patients who have been taking betahistine for a longer duration is necessary to understand the long-term effects of this medication on the VOR system.
